# Olaparib tolerability and common adverse-event management in patients with metastatic castration-resistant prostate cancer: Further analyses from the PROfound study

**DOI:** 10.1016/j.ejca.2022.04.016

**Published:** 2022-05-19

**Authors:** Guilhem Roubaud, Mustafa Özgüroğlu, Nicolas Penel, Nobuaki Matsubara, Niven Mehra, Michael P. Kolinsky, Giuseppe Procopio, Susan Feyerabend, Jae Young Joung, Gwenaelle Gravis, Kazuo Nishimura, Craig Gedye, Charles Padua, Neal Shore, Antoine Thiery-Vuillemin, Fred Saad, Robbert van Alphen, Michael A. Carducci, Chintu Desai, Neil Brickel, Christian Poehlein, Paula Del Rosario, Karim Fizazi

**Affiliations:** a Department of Medical Oncology, Institut Bergonié, Bordeaux, France; b Department of Internal Medicine, Division of Medical Oncology, Cerrahpașa School of Medicine, Istanbul University-Cerrahpașa, Istanbul, Turkey; c Lille University and Centre Oscar Lambret, Lille, France; d National Cancer Center Hospital East, Chiba, Japan; e Radboud University Medical Center, Nijmegen, the Netherlands; f Department of Oncology, University of Alberta, Cross Cancer Institute, Edmonton, Alberta, Canada; g Medical Oncology Department, Fondazione IRCCS Istituto Nazionale Dei Tumori, Milan, Italy; h Urologic Oncology, Studienpraxis Urologie, Nürtingen, Germany; i Center for Prostate Cancer, National Cancer Center, Goyang, South Korea; j Institut Paoli-Calmettes, Marseilles, France; k Department of Urology, Osaka International Cancer Institute, Osaka, Japan; l Calvary Mater Newcastle, Waratah, Australia; m Cetus Medicina Oncológica, Betim, Brazil; n Carolina Urologic Research Center, Myrtle Beach, SC, USA; o Medical Oncology Unit, CHU Besançon, Besançon, France; p Centre Hospitalier de L’Universite de Montreal, Montreal, Canada; q Elisabeth-Tweesteden Ziekenhuis, Tilburg, the Netherlands; r Hopkins Kimmel Cancer Center, Maryland, USA; s AstraZeneca, Cambridge, UK; t Merck & Co., Inc., Kenilworth, NJ, USA; u Department of Cancer Medicine, Institut Gustave Roussy, University of Paris Saclay, Villejuif, France

**Keywords:** PROfound, Metastatic castration-resistant prostate cancer, Olaparib, Safety, Adverse-event management

## Abstract

**Background:**

Based on PROfound, olaparib is approved for patients with metastatic castration-resistant prostate cancer following disease progression on at least enzalutamide or abiraterone and who carry relevant alterations in DNA repair genes. To facilitate continued olaparib treatment as long as the patient derives benefit, we describe further safety assessments from PROfound focusing on the four most common adverse events (AEs) and events of special interest.

**Methods:**

Patients were randomized (2:1) to olaparib tablets (300 mg bid) or control (enzalutamide or abiraterone) until disease progression or unacceptable toxicity. Safety was assessed through AE reporting and laboratory assessments. Safety data were also collected from all patients in the control group who experienced radiographic disease progression and subsequently crossed over to olaparib treatment.

**Results:**

256 patients received olaparib and 130 control. Incidence rates for the four most commonly occurring AEs in the olaparib group (all-causality) were anaemia 50%, nausea 43%, fatigue/asthenia 42% and decreased appetite 31%. All were mostly Grade 1 and 2 and all peaked within the first 2 months of treatment as the events were managed where appropriate, primarily with dose interruptions or dose reductions. The extent of bone metastases at baseline or prior taxane use was not associated with the rate of anaemia. Pneumonitis was reported in 2% and 1.5% of patients in the olaparib and control groups, respectively, and one patient (0.4%) in the olaparib group experienced an event of MDS/AML after a 30-day follow-up period. Venous thromboembolic events occurred in 8% of olaparib and 3% of control patients.

**Conclusions:**

The four most common AEs observed in PROfound were generally manageable without the need for treatment discontinuation, allowing patients to remain on treatment for as long as they were deriving clinical benefit.

**ClinicalTrials: gov registration number:**

NCT02987543.

## 1. Introduction

Olaparib is a poly (ADP-ribose) polymerase (PARP) inhibitor approved for the treatment of specific patient populations with four tumour types: ovarian, breast, pancreatic and most recently, prostate cancer [1,2]. Olaparib’s first approval was in December 2014 for BRCA-altered advanced ovarian cancer [3] and there is now extensive experience of olaparib use in the clinical environment.

In the phase III PROfound trial, olaparib significantly prolonged radiographic progression-free survival (median 7.4 versus 3.6 months; hazard ratio [HR], 0.34; P < 0.001) and overall survival (median 19.1 versus 14.7 months; HR, 0.69; P = 0.02) compared with physician’s choice of enzalutamide or abiraterone (control) in patients with metastatic castration-resistant prostate cancer and alterations in *BRCA1*, *BRCA2* and *ATM* whose disease had progressed on a prior next-generation hormonal agent, and which included patients who crossed over to olaparib treatment at disease progression [4,5]. PROfound was a pivotal study in the treatment of mCRPC and resulted in the approval of olaparib in the USA and Europe in May and November 2020, respectively, for the treatment of patients with mCRPC and specific genomic alterations associated with DNA defect repair and following progression on at least prior next-generation hormonal agents [6,7].

Consistent with the established safety profile of olaparib across the various tumour types, the treatment-emergent adverse events (TEAE) most frequently observed in the olaparib group of PROfound irrespective of causality were anaemia, nausea, fatigue/asthenia and decreased appetite. Anaemia was also the event responsible for the highest number of patients (7%) discontinuing olaparib treatment [8].

We present further safety and tolerability data from PROfound with the aim of increasing the understanding of the clinical management of the most common adverse events (AEs) to aid decision-making, reduce discontinuations and facilitate continued olaparib therapy for as long as the patient is deriving benefit. Specifically, we report on the incidence, onset and duration of the four most common AEs and their clinical management. Additionally, we examine incidences of two AEs of special interest as highlighted in the prescribing information [1,2]: pneumonitis and myelodysplastic syndrome (MDS)/acute myeloid leukaemia (AML). We also report on the thromboembolic disorders observed in PROfound because a significant risk of venous thromboembolic events is inherently present in metastatic castration-resistant prostate cancer (mCRPC) associated with the use of continuous androgen deprivation therapy (ADT) [5].

## 2. Methods

### 2.1. Study design, participants and outcomes

The PROfound study design, eligibility criteria, methods and patient disposition have previously been published in detail [4,8]. In brief, eligible patients with mCRPC and disease progression on a prior next-generation hormonal agent (eg enzalutamide or abiraterone) were randomized 2:1 to olaparib tablets (300 mg twice daily [bid]) or a control of physician’s choice of enzalutamide (160 mg/day) or abiraterone (1000 mg/day plus prednisone at 5 mg bid). Patients included in Cohort A had at least one alteration on somatic tumour-based testing in *BRCA1*, *BRCA2* or *ATM*, and Cohort B patients had alterations in or ≥1 of 12 other prespecified genes with a direct or indirect role in homologous recombination repair: *BARD1*, *BRIP1*, *CDK12*, *CHEK1*, *CHEK2*, *FANCL*, *PALB2*, *PPP2R2A*, *RAD51B*, *RAD51C*, *RAD51D* and/or *RAD54L*.

The primary endpoint of radiographic progression-free survival in Cohort A was assessed by blinded independent central review. Safety and tolerability in the overall population (Cohorts A and B) were assessed through adverse-event reporting according to the Common Terminology Criteria Adverse Event (CTCAE v4.03). Patient safety assessments were scheduled on Day 1 of treatment and at weeks 4, 8, 12, 16, 20, 24 and then every 8 weeks and also on discontinuation. A safety follow-up was conducted 30 days after the last dose and any new event identified was followed to resolution. Safety data were also collected from all patients in the control group who experienced radiographic disease progression and subsequently crossed over to olaparib treatment.

With specific relevance to the data we present, AEs related to olaparib could be managed by dose interruptions and reductions. Dose interruptions were allowed for a maximum of 4 weeks on each occasion and if the interruption was longer, the study team was required to be informed. For dose reductions, and in alignment with clinical practice guidelines developed for ovarian cancer at the time of study initiation, olaparib could be reduced from 300 mg bid to 250 mg bid and then to 200 mg bid if further reduction was required. If the reduced dose of 200 mg bid was not tolerable, no further reduction was allowed and study treatment was discontinued. As described in the study protocol, no routine prophylactic anti-emetic treatment for nausea was required at the start of study treatment and nausea could be managed by anti-emetic treatment at first onset and as required afterwards, in accordance with local treatment practice. Alternatively, patients could also take olaparib tablets with a light meal or snack.

For the management of anaemia (enrolment criteria was Hb ≥ 10.0 g/dL with no blood transfusions in the past 28 days), common treatable causes such as iron, vitamin B12 or folate deficiencies and hypothyroidism could be appropriately managed after an investigation into the cause. Actions to be taken for the management of Grade 2 and Grade 3 anaemia, such as dose reduction or interruption, and blood transfusion are summarized in Supplementary Table 1.

Additional exploratory post-hoc analyses (reported here) include investigating the rates and severity of anaemia with respect to the extent of bone metastases at baseline (<10 or ≥10 lesions), prior taxanes use and type of alteration (germline or somatic) and further investigation into incidences of thromboembolic events because mCRPC carries a naturally higher risk of these events occurring [5].

The trial was performed in accordance with the principles of the Declaration of Helsinki, the International Conference on Harmonisation Good Clinical Practice guidelines, and the AstraZeneca and Merck policies on bioethics. All the patients provided written informed consent.

### 2.2. Statistical analysis

The safety population comprised all patients in receipt of at least one dose of study treatment. Safety data were analysed descriptively (data cut-off, 20 March 2020). Anaemia is reported as a ‘grouped term’ based on Medical Dictionary for Regulatory Activities (MedDRA)-preferred terms and includes anaemia, decreased haemoglobin level, decreased red-cell count, decreased haematocrit level, erythropenia, macrocytic anaemia, normochromic anaemia, normochromic normocytic anaemia and normocytic anaemia. Similarly, pneumonitis is reported as a grouped term and includes pneumonitis, interstitial lung disease and radiation pneumonitis. For reporting of the number of patients with AEs, patients with multiple events in the same preferred term are counted only once in that preferred term. Patients with events in more than one preferred term are counted once in each of those preferred terms.

## 3. Results

### 3.1. Patients and treatment

The PROfound safety population consisted of 256 patients in the olaparib group and 130 in the control group (one patient randomized to control did not receive study treatment). Baseline characteristics were generally balanced between the olaparib group and the control, including the use of prior next-generation hormonal and taxane agents (a stratification factor) and baseline Hb (12.3 g/dL in the olaparib group and 11.9 g/dL in the control). Patient characteristics at baseline are shown in Supplementary Table 2. Of the 131 patients randomized to the control group, 83 (63%) crossed over at confirmed disease progression to receive olaparib. The median total treatment duration for olaparib was 7.6 months (range 0.03–28.9) and for the control 3.9 months (0.6–29.1). For patients who crossed over, the median duration of treatment was 4.8 months (range, 0.2 to 28.9).

### 3.2. Adverse events

**Fig. 1.**
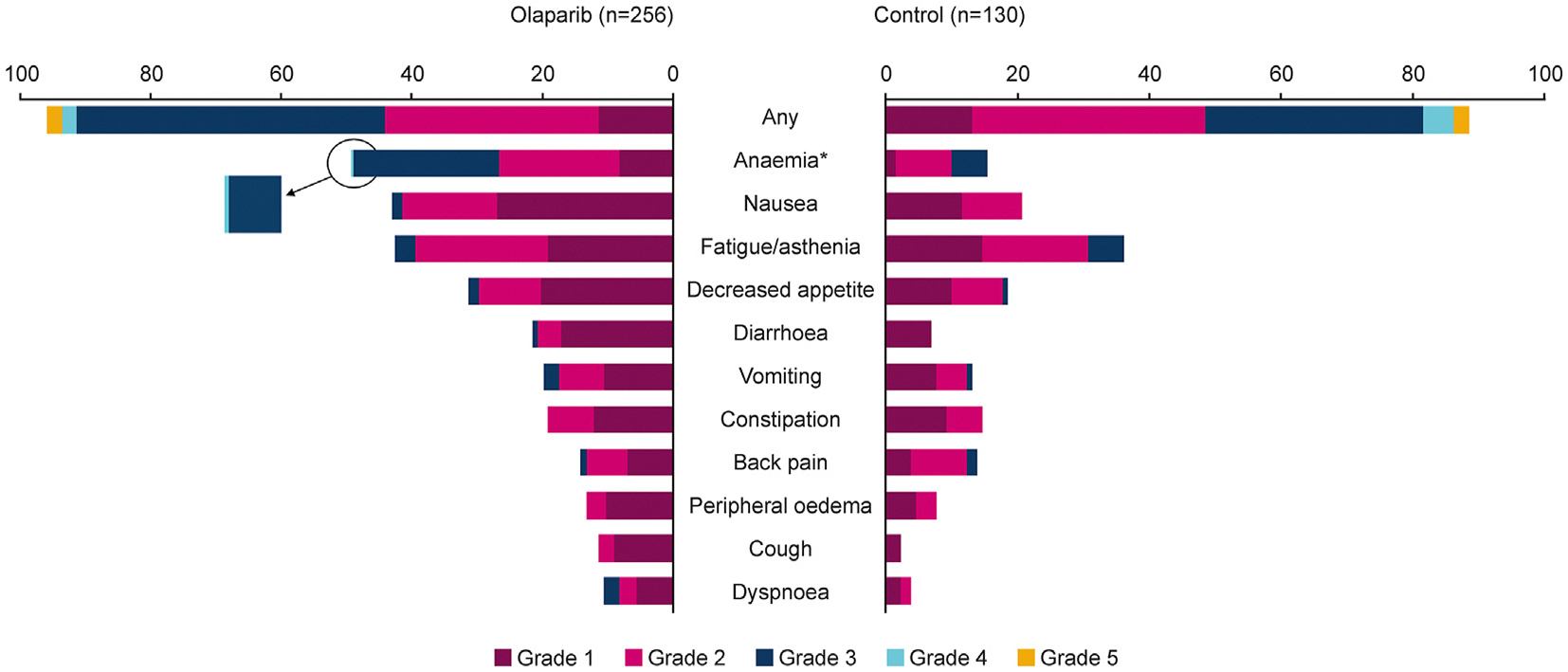
Most common AEs (all causality) occurring in >10% of patients in either treatment group (by CTCAE grade 4.03) For the most common AEs occurring in >10% of patients, in the olaparib group one Grade 4 AE was observed for anaemia and there were no Grade 5 AEs. In the control group, there were no AEs above Grade 3. *Anaemia is reported as a ‘grouped term’ based on MedDRA-preferred terms and includes anaemia, decreased haemoglobin level, decreased red-cell count, decreased haematocrit level, erythropenia, macrocytic anaemia, normochromic anaemia, normochromic normocytic anaemia, and normocytic anaemia.

**Fig. 2.**
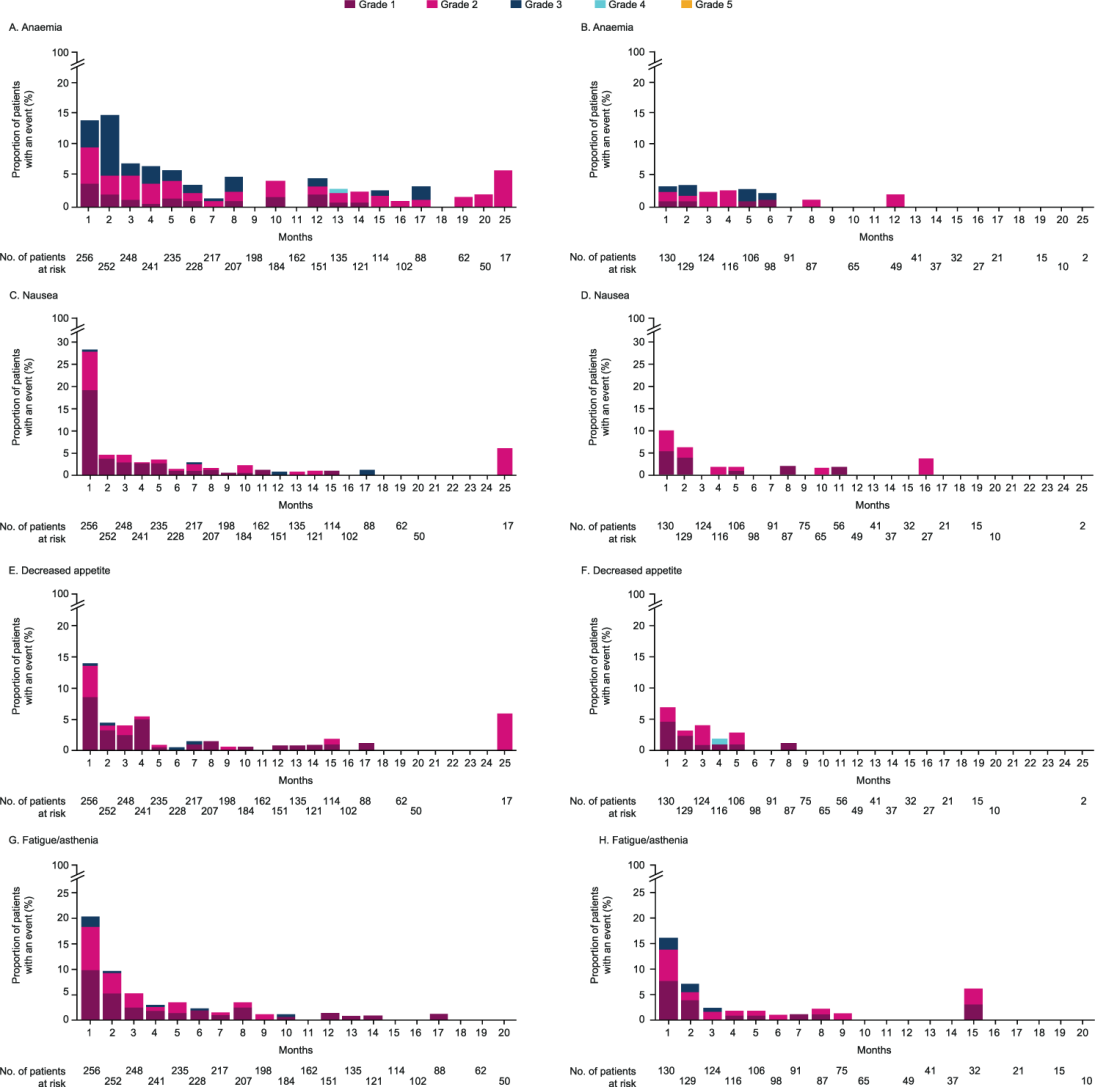
Incidence by month and grade for the four most common AEs (all causality) over the course of olaparib treatment (A, C, E and G) and control treatment (B, D, F and H) These AEs are expected to have been actively managed where appropriate during treatment. *Anaemia is reported as a ‘grouped term’ based on MedDRA-preferred terms and includes anaemia, decreased haemoglobin level, decreased red-cell count, decreased haematocrit level, erythropenia, macrocytic anaemia, normochromic anaemia, normochromic normocytic anaemia and normocytic anaemia.

In total, 246 (96%) patients from the safety population in the olaparib group experienced an AE (all-causality) compared with 115 (88%) for the control group. The most frequently observed AE in the olaparib group was anaemia (50% of all patients) of which 23% were Grade ≥3. For the control group, 15% of all patients were observed with anaemia, 5% at Grade ≥3. Nausea was observed in 43% of all patients in the olaparib group (2% at Grade ≥3) and 21% of all patients in the control (all at Grade 1 or 2). Fatigue/asthenia was observed in 42% of all patients in the olaparib group of which 3% were Grade ≥3, and in 33% of all patients in the control, with 5% of patients at Grade ≥3. Decreased appetite occurred in 31% of all patients in the olaparib group of which 2% were Grade ≥3, and in 19% of all patients in the control, of which 1% were Grade ≥3 (Fig. 1). AEs mainly occurred within the first 2 months of olaparib treatment (Fig. 2A, C, E and G) before beginning a trend downwards in incidence numbers and severity as they would have expected to have been actively managed where appropriate during treatment. A similar pattern was observed in the control group (Fig. 2B, D, F and H).

In the olaparib group, the incidence of anaemia peaked at month 2 reducing by 53% at month 3 and the number of patients with Grade ≥3 anaemia also decreased by 80%. In the control population, occurrences of anaemia were observed to be at a smaller and more consistent level than with olaparib (Fig. 2A and B). Nausea in the olaparib group peaked in month 1 (72 patients) and resolved quickly with a substantial drop of 85% by month 2. In the control group, a similar pattern was observed of an early peak and downward trend (Fig. 2C and D). Decreased appetite also peaked in the olaparib group during month 1 declining by 69% at month 2 (Fig. 2E and F). Fatigue/asthenia similarly peaked at month 1 declining by 54% to 24 patients at month 2 and continuing a downwards trend. A similar pattern was observed in the control group, with the exception of a single isolated event occurring in one of 32 patients (3%) at month 15 after 5 months of no events occurring (Fig. 2G and H).

**Fig. 3.**
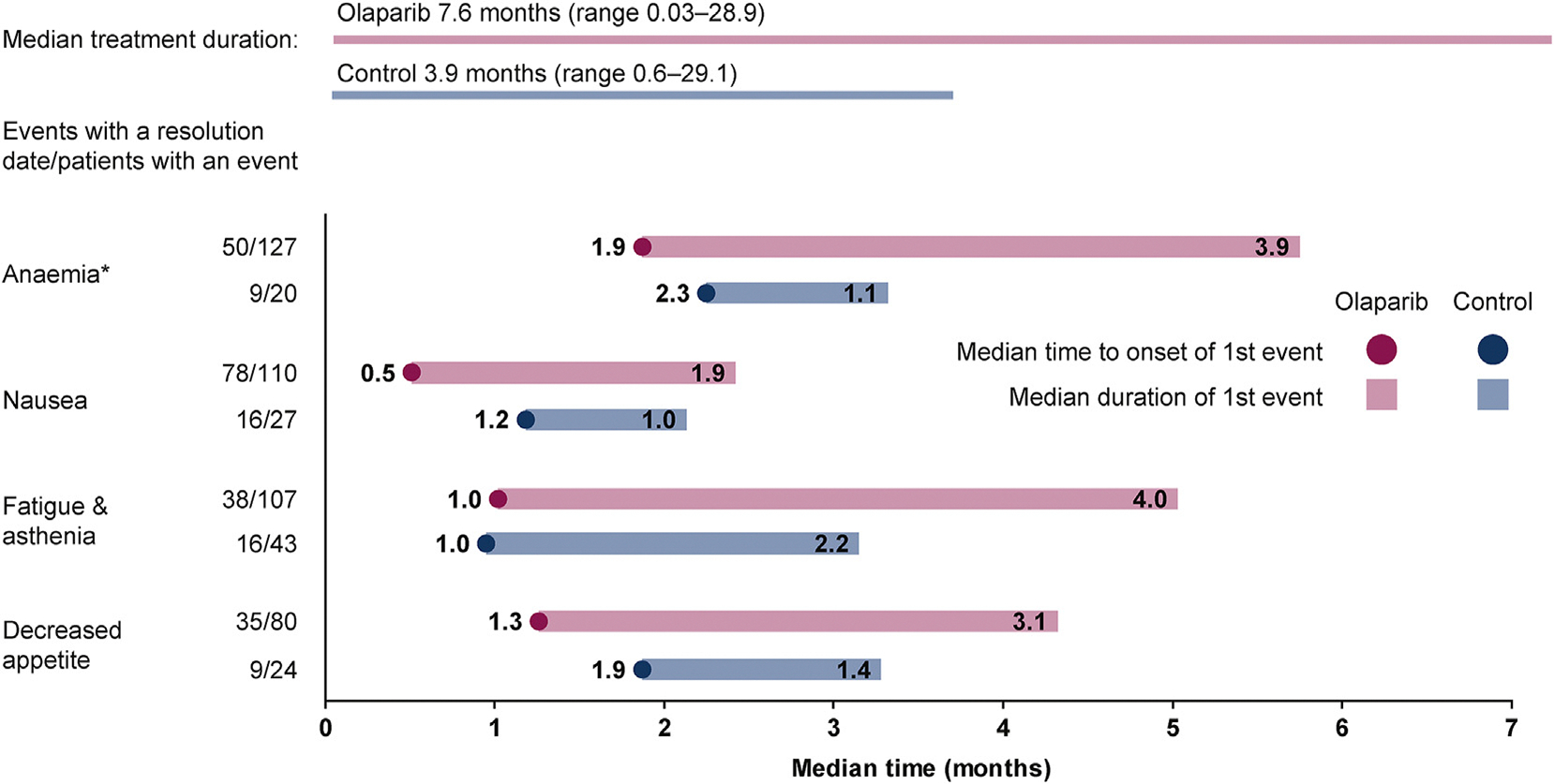
Median time to first onset and duration of the most common AEs (all causality) in patients who experienced the event. These AEs are expected to have been actively managed where appropriate during treatment. *Anaemia is reported as a ‘grouped term’ based on MedDRA-preferred terms and includes anaemia, decreased haemoglobin level, decreased red-cell count, decreased haematocrit level, erythropenia, macrocytic anaemia, normochromic anaemia, normochromic normocytic anaemia, and normocytic anaemia.

**Fig. 4.**
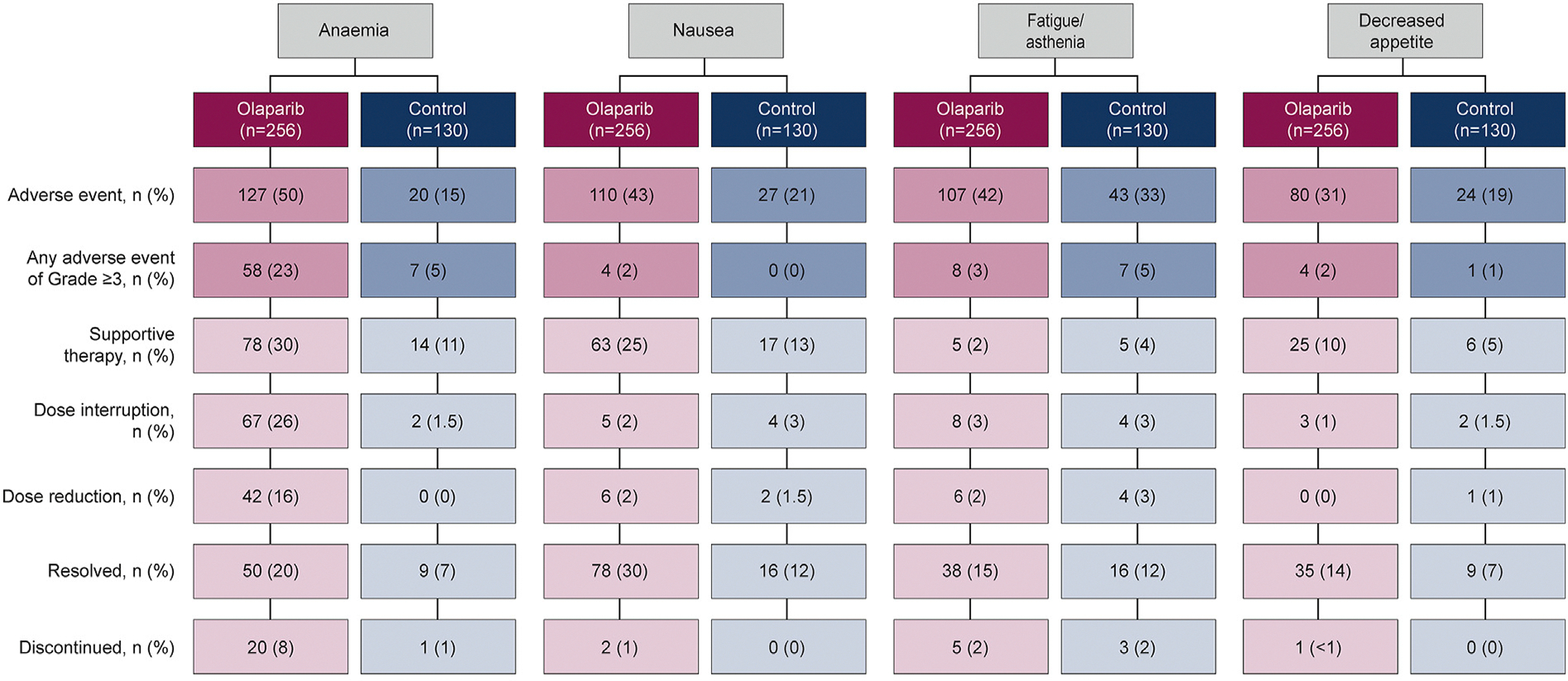
Management of the four most common AEs (all causality) . Patients with multiple AEs leading to a reduction in treatment were counted once for each preferred term. *Anaemia is reported as a ‘grouped term’ based on MedDRA-preferred terms and includes anaemia, decreased haemoglobin level, decreased red-cell count, decreased haematocrit level, erythropenia, macrocytic anaemia, normochromic anaemia, normochromic normocytic anaemia, and normocytic anaemia. ^†^In accordance with local treatment practice.

For patients receiving olaparib, nausea had the shortest median time to first onset (0.5 months) and anaemia the longest (1.9 months). Nausea also had the shortest median duration of first event (1.9 months) and fatigue/asthenia the longest (4.0 months). In the control group, all four AEs had a shorter median duration of first event and later time to onset than for the olaparib group, with the exception of fatigue/asthenia, which shared the same time to first onset, 1 month (Fig. 3). With regard to AE management, for the patients with anaemia (n = 127) in the olaparib group (n = 256), the most common treatment option was supportive therapy (78/256, 30%), followed by dose interruptions (67/256, 26%) and dose reductions (42/256, 16%). The anaemia was resolved in 50/256 (40%) patients and 20 (8%) discontinued. Supportive therapies consisted of 66 (52%) patients receiving between 1 and 20 (median 3) blood transfusions (red blood cell products and whole blood transfusions) as a concomitant medication and 13 (10%) receiving an erythropoiesis-stimulating agent; in total, 71 (56%) patients had a blood transfusion and/or anti-anaemic treatment. In the 110/256 (43%) patients experiencing nausea, 63/110 (57%) were treated with supportive therapy and it was resolved in 78 (71%) patients. Dose interruptions and reductions, and discontinuations were all notably higher for anaemia than for nausea, fatigue/asthenia and decreased appetite (Fig. 4).

**Fig. 5.**
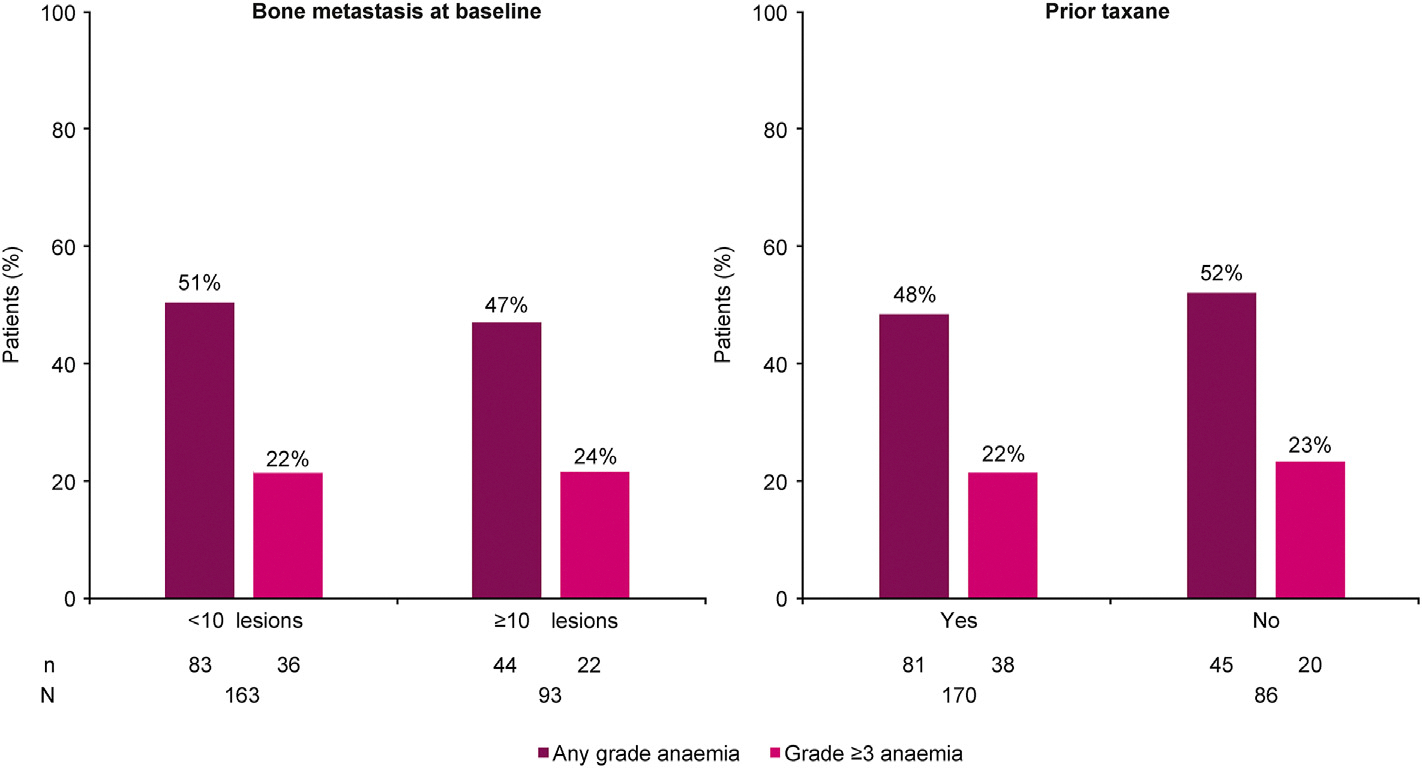
Frequency of anaemia with olaparib compared to control arm in patients with more bone metastases or who received prior taxanes.

The impact of bone metastasis on anaemia in patients who received olaparib was assessed by dividing patients into two groups based on the number of baseline lesions (<10 or ≥10) (Fig. 5). The incidence of anaemia (all grades and Grade≥3) was similar between the two groups: 51% versus 47% and 22% versus 24%, respectively. With regard to treatment management between the two groups (<10 or ≥10), the number of patients with anaemia who had a dose interruption was 28% and 24% and a dose reduction of 18% and 13%, respectively. Treatment was discontinued in 6% of patients in the <10 bone lesions group and 11% in the ≥10 group. The risk of anaemia was also investigated in patients who had or had not received a prior taxane, and again the risk of anaemia was numerically similar between the two groups: 48% versus 52% (Fig. 5). Additionally, among 42 patients in the olaparib arm identified with either a germline *BRCA1* or *BRCA2* alteration, 26% of these patients had grade ≥3 anaemia. For the 33 patients in the olaparib arm identified with a somatic *BRCA1* or *BRCA2* alteration, 18% had grade ≥3 anaemia.

### 3.3. AEs of special interest and venous thromboembolic events

Pneumonitis (all-causality) was reported in five patients (2%) in the olaparib group and two (1.5%) in the control arm. For the olaparib group, one patient’s (Grade 1 pneumonitis) study treatment continued without change and the event was ongoing at data cut-off. Events in a second patient (Grade 2) resolved after 10 days and a third patient (Grade 2) had their treatment interrupted due to the event that was ongoing at data cut-off. A fourth patient’s (Grade 3) study treatment was also interrupted with the event ongoing at data cut-off. The final patient (Grade 3) discontinued study treatment as a result of the pneumonitis event which resolved after 46 days. In the control group, one patient (Grade 1) had no treatment change and the event was ongoing at data cut-off and the remaining patient’s (Grade 2) event resolved after 84 days. No pneumonitis event in the olaparib or control group had a fatal outcome.

One patient (0.4%) in the olaparib group reported an event of MDS/AML (reported term: leukaemia) that occurred after the 30-day follow-up period. The patient had received olaparib until Day 478 due to an AE of grade 3 thrombocytopenia and permanently discontinued study treatment on Day 505. The patient did not receive any subsequent therapy for prostate cancer. On Day 558, an SAE of grade 5 leukaemia was reported and the patient died due to this event on the same day. The investigator considered the event of leukaemia to not be causally related to treatment with olaparib. Venous thromboembolic events occurred in 20 (8%) patients in the olaparib group and four (3%) control patients. Three patients had treatment interruptions due to these events (two in the olaparib group and one control) and one patient in the olaparib group discontinued. Of the 20 patients in the olaparib arm who experienced a thromboembolic event, 12 (60%) previously received a blood transfusion or other anti-anaemic treatment. For the 236 patients in the olaparib arm with no thromboembolic event, 59 (25%) received a transfusion or other anti-anaemic treatment. In the control arm, 4 patients experienced a thromboembolic event, none of whom received a blood transfusion, and of the 126 patients who had no thromboembolic event, 14 (11%) received a transfusion or other anti-anaemic treatment. Pulmonary embolism was the thromboembolic event observed most frequently - in 12 (5%) patients in the olaparib group (5 at Grade 1 or 2, 7 at Grade ≥3) and 1 patient (1%) in the control group (Grade ≥3) (Table 1). Time to onset for pulmonary embolism in the olaparib group ranged from 0.2 to 11.3 months.

## 4. Discussion

We report here further investigations from PROfound into the four most commonly occurring AEs associated with monotherapy olaparib treatment in patients with mCRPC, with the clinical goal of aiding decision making and facilitating continued olaparib therapy for as long as the patient is deriving benefit.

The four most commonly observed AEs in olaparib-treated patients in our study (anaemia, nausea, fatigue/asthenia and decreased appetite) have also been reported as among the most commonly observed AEs in studies of olaparib in other tumour types, including breast [9–11], ovarian [12–17], and pancreatic cancers [18]. For example, the incidence of anaemia was 23% in the Study 19 ovarian cancer study [12], 27% in the POLO pancreatic cancer study [18], 40% in the OlympiAD breast cancer study [10] and 39%, 44%, 51% and 39% in the SOLO1 [13], SOLO2 [14], SOLO3 [15] and OPINION [16] ovarian cancer studies, respectively. The incidence we report for the PROfound study was 50%, although it should be noted that anaemia AEs reported were sometimes based on grouped terms which can differ between studies, hence comparing incidences of anaemia between studies can be unreliable. Nausea incidences ranged from 45% in the POLO pancreatic cancer study [18] to 77% in the SOLO1 ovarian cancer study [13]) and was 43% for the PROfound study we report. Similarly, the incidence of fatigue/asthenia ranged from 39% in the OlympiAD breast cancer study [10] to 66% in the SOLO2 ovarian cancer study [14], and was 42% for PROfound. Finally, the incidence of decreased appetite was 25% in the POLO pancreatic cancer study [18] and 31% in PROfound.

Our study showed that all four of the most commonly observed AEs in olaparib-treated patients (anaemia, nausea, fatigue/asthenia and decreased appetite) had early onset and peaked within the first month of treatment with the exception of nausea, which peaked during the second month. All then declined in incidence and severity over time, and all were typically resolved within the first 6 months of treatment. This is likely to be because of early intervention with appropriate supportive therapies (in accordance with local treatment practice) along with dose interruption or reduction, allowing the majority of patients experiencing AEts to continue without the need for treatment discontinuation. It is not thought that the decline in events was due to the development of tolerance to the toxicity as seen, for example, with opioid treatment [19]. Discontinuation of olaparib treatment from the four AEs was infrequent (11% of the total patients receiving olaparib) and was highest for anaemia (8% of patients who received olaparib). No deaths were reported attributed to the four most common AEs.

The most common supportive therapies for anaemia (52% of patients experiencing anaemia) were blood transfusion (patient’s red blood cell products and whole blood transfusions) and erythropoiesis-stimulating agents (10% of patients), primarily epoetin alpha and darbepoetin alpha. Although incidences of anaemia peaked in month 2, there were still occurrences throughout the 20-month monitoring period, reinforcing the need for continual patient monitoring throughout the duration of olaparib treatment, consistent with the current recommended monitoring frequencies for haematological AEs – at baseline and monthly thereafter according to olaparib prescribing information [1,2]. Currently there are no specific recommendations in the olaparib patient information leaflet (PIL) [20], nor the EMA summary of product characteristics (SPC) [21] and FDA prescribing information [22] documents for the use of erythropoiesis-stimulating agents to treat anaemia, nor are there any similar recommendations included in the PIL/SPC/prescribing information documents for other licenced PARP inhibitors, rucaparib [23,24], talazoparib [25,26] or niraparib [27,28], in their respective approved indications.

In the mCRPC setting, up to 90% of patients have bone involvement [29], and poor bone health can be also exacerbated by the substantial increase in bone turnover resulting from ADT [30,31]. In patients in the olaparib group in PROfound, incidences of anaemia analysed according to the number of bone metastases at baseline (<10 bone lesions compared with ≥10) suggested no increased risk of anaemia in patients with more baseline bone metastases than those with less. This observation is also supported when the amount of bone metastases at baseline and rates of anaemia are considered for other pivotal olaparib studies on other solid tumours (see Supplementary Table 3 and accompanying text). Also, from our analysis, we found no evidence of an increased risk of anaemia in olaparib-treated patients who have received prior taxane therapy compared with those who did not. In the olaparib arm, there was a slightly higher proportion of patients with grade ≥3 anaemia among those with a germline *BRCA* alteration compared to among those with a somatic alteration (26% versus 18%, respectively), although the small patient numbers involved prevent any robust inferences to be made.

The mechanism by which PARP inhibitors lead to haematological AEs, particularly anaemia, remains to be elucidated. Preclinical evidence suggests that PARP-2 plays a pivotal role in erythroid differentiation and its deletion leads to extravascular haemolytic anaemia suggesting that PARP inhibitors may impact haematopoiesis, which may explain the haematological AEs observed in these studies [32,33]. Myelosuppression is a class effect that has been observed in clinical trials of other PARP inhibitors in patients with mCRPC following prior next-generation hormonal agent and taxane therapy. For example, in the phase II TRITON2 study evaluating rucaparib for the treatment of mCRPC in a genomically selected patient population, the incidence of anaemia/decreased haemoglobin was 44%. Additionally, the most frequent Grade ≥3 TEAE was anaemia/decreased haemoglobin (25%). Overall, 32 patients (28%) in TRITON2 received ≥1 blood transfusion [34]. Similarly for niraparib, in the phase II GALAHAD study in patients with mCRPC and DNA-repair gene defects, the most common Grade ≥3 TEAE was anaemia (29% of patients) [35]. Also, for talazoparib in the phase II TALAPRO1 study in patients with mCRPC and DNA damage repair mutations, anaemia was also the most common TEAE [36]. As far as we are aware, a direct comparison between different PARP inhibitors regarding myelosuppressive events in mCRPC has not been investigated though under clinical trial conditions.

Nausea is a commonly reported AE in patients receiving any PARP inhibitor and management strategies, based on real-world experiences, include advising patients to consume food before taking their dose, using anti-emetics for the first month followed dose reductions, if needed, in patients with Grade ≤2 nausea [37,38]. For patients with Grade ≥3 nausea, dose interruptions and anti-emetics are required until the nausea improves, and then treatment can be restarted along with prophylactic anti-emetic use [37]. Although prophylactic use of anti-emetics is not required at the start of treatment (according to current PARP inhibitor labels), their use should be considered to reduce the potential for nausea. In their recently published clinical practice guidelines on anti-emesis, the National Comprehensive Cancer Network (NCCN) considers olaparib has a moderate to high risk of emesis, and so recommend the use of 5-HT3 receptor antagonists such as ondansetron, dolasetron or granisetron for prophylaxis of nausea and vomiting. The H2 blocker lorazepam or a proton pump inhibitor may also be added (either alone or in combination) [39]. There was no specific guidance in the PROfound study protocol for olaparib dose reduction, interruption or discontinuation and anti-emetic treatment, for nausea. Ultimately, decisions on the management of nausea, including the restarting dose after nausea, rested on the clinical judgement of the investigator.

In patients presenting with fatigue, some parameters potentially involved need to be raised including anaemia or other organ dysfunction, mainly cardiac insufficiency, or hypothyroïdy. Also, potential drug–drug interactions with concomitant treatments have to be investigated. Fatigue has then to be assessed and managed by a multidisciplinary team of supportive care including adapted physical activity. Decreased appetite and potential weight loss sometimes associated with sarcopenia have also to be assessed with an individual dietetic counselling. Fatigue as well as decreased appetite may be caused or associated with depression and require psychological counselling. Ideally, all these supportive cares should be initiated prior to the beginning of PARP inhibitors, in order to optimize their dosing.

In PROfound, the incidence of pulmonary embolism (all causality) was higher in the olaparib group than in the control group; 12 patients (5%) versus 1 patient (<1%), respectively (Table 1). All patients in PROfound had mCRPC which has progressed on prior next-generation hormonal agent treatment and at this advanced stage of the disease a significant risk of venous thromboembolic events is inherently present [5]. Patients had also received treatment with ADT prior to the trial, and unlike previous studies of olaparib in other solid tumours, continuous ADT during the trial, both of which can contribute further to an increased risk of thromboembolic events [40], as may the administration of erythropoiesis-stimulating agents for cancer-associated anaemia [41].

The increased baseline risk of a thromboembolic event, the almost two-fold longer exposure in the olaparib group, the lack of imbalance in venous thrombosis events and the short duration of follow-up available make it difficult to interpret the observed imbalance in pulmonary embolism/embolic events between the study groups as no causal association was established. Additionally, pulmonary embolism was not observed to be an AE of note in previous olaparib studies in different solid tumours [9,13,18]. Caution is also advised in the interpretation of thromboembolic events with regard to a potential connection between the use of blood transfusions or other anti-anaemic treatment and thromboembolic events due to the small numbers of patients with a thromboembolic event in the PROfound patient population. With regard to other PARP inhibitor studies, in TALAPRO-1 study, 6.3% of 128 patients receiving talazoparib experienced pulmonary embolism [42]. With respect to the two AEs of special interest for olaparib (pneumonitis and of MDS/AML), the additional data from PROfound we report does not change the assessment of causal association between olaparib treatment and the two other AEs of special interest, which is based on programme-wide data, i.e. patients receiving olaparib and experiencing these events should, once the event is confirmed, be discontinued as per the prescribing information.

## 5. Conclusions

Overall, and with the caveat that a formal cross-trial comparison has not been conducted, the safety profile of olaparib in PROfound was similar to that previously characterized in patients with other solid tumours (breast, ovarian and pancreatic cancer) and treated with olaparib, and on which there is a wealth of clinical experience available since the first approval of olaparib in ovarian cancer in 2014. The most common AEs in PROfound were anaemia, nausea, decreased appetite and fatigue/asthenia, which is consistent with observations from other clinical studies on other tumour types and real-world evidence. Incidences of these four AEs peaked within the first 2 months of treatment and were generally manageable through dose modifications and supportive therapies without the need for treatment discontinuation, allowing patients to remain on treatment for as long as they were deriving clinical benefit.

## Supplementary Material

Appendix Materials

## Figures and Tables

**Table 1:** Summary of venous thromboembolic events by preferred term with exposure-adjusted rates.

	Olaparib 300 mg bid (n = 256)		Control (n = 130)	
	No. (%) of patients	Event rate (per 1000 patient years)^a^	No. (%) of patients	Event rate (per 1000 patient years)*

**Patients with a thromboembolic event**	**20 (7.8)**	**101.29**	**4 (3.1)**	**65.46**
Pulmonary embolism	12 (4.7)	58.97	1 (0.8)	16.31
Deep vein thrombosis	4 (1.6)	19.08	2 (1.5)	32.63
Embolism	4 (1.6)	19.01	0	0
Mesenteric vein thrombosis	1 (0.4)	4.74	0	0
Vena cava thrombosis	1 (0.4)	4.70	0	0
Venous thrombosis	1 (0.4)	4.73	1 (0.8)	16.31
Thrombosis	0	0	1 (0.8)	16.30

AE, adverse event; bid, twice daily; NHA, next-generation hormonal agent.

a Number of patients with that AE divided by the sum of the duration of therapy (for patients without the AE) and the time to the AE (for patients with the AE) in each group multiplied by 1000. The number of years at risk of AE across patients on olaparib is 197.45 years and Investigators Choice of NHA is 61.11 years. Includes AEs with an onset date on or after the date of first dose and up to and including 30 days following discontinuation of randomized treatment or the day before switching to olaparib.
