# Supplementary material for: Olaparib tolerability and common adverse-event management in patients with metastatic castration-resistant prostate cancer: Further analyses from the PROfound study
Source: Eur J Cancer. Author manuscript; Available in PMC 2025 Mar 18. (PMC11919091; doi:10.1016/j.ejca.2022.04.016)
Supplement: Appendix Materials [file NIHMS2059916-supplement-Appendix_Materials.docx]

# Supplement to:

**Olaparib tolerability and common adverse-event management in patients with metastatic castration-resistant prostate cancer: further analyses from the PROfound study**

Guilhem Roubaud, Mustafa Özgüroğlu, Nicolas Penel, Nobuaki Matsubara, Niven Mehra, Michael Kolinsky, Giuseppe Procopio, Susan Feyerabend, Jae Young Joung, Gwenaelle Gravis, Kazuo Nishimura, Craig Gedye, Charles Padua, Neal Shore, Antoine Thiery-Vuillemin, Fred Saad, Robbert van Alphen, Michael Carducci, Chintu Desai, Neil Brickel, Christian Poehlein, Paula Del Rosario, Karim Fizazi

**Contents**

**Page**

Supplementary Table 1: Management of anaemia 2

Supplementary Table 2: Patient characteristics at baseline 3

Supplementary Table 3: Summary of exposure data, baseline bone metastases and anaemia AEs across olaparib pivotal studies 4

**Supplementary Table 1**

Management of anaemia

| **Haemoglobin** | **Action to be taken** |
| --- | --- |
| Hb < 10 *but* ≥8 g/dL  (CTCAE Grade 2) | Give appropriate supportive treatment, continue to monitor and investigate causality   - For *first* incidence: investigator judgement to continue olaparib with or without supportive treatment (eg transfusion) or interrupt dose for a maximum of 4 weeks - For *repeat* incidence (after recovery of first event) - If Hb < **10** but ≥ 9 g/dL investigator judgement to continue olaparib with supportive treatment (eg transfusion) or dose interrupt (for max of 4 weeks) and upon recovery, dose reduction may be considered (to **250 mg twice daily** as a first step and to **200 mg twice daily** as a second step) - If Hb < **9** but ≥ **8 g/dL**, dose interrupt (for max of 4 weeks) until Hb ≥ 9 g/dL and upon recovery, dose reduction may be considered (to **250 mg twice daily** as a first step and to **200 mg twice daily** as a second step) |
| Hb < 8 g/dL  (CTCAE Grade 3) | Give appropriate supportive treatment (eg transfusion), continue to monitor and investigate causality  Interrupt olaparib for a maximum of 4 weeks until improved to  Hb ≥ 9 g/dL  Upon recovery dose, reduce olaparib to **250 mg twice daily** as a first step and to **200 mg twice daily** as a second step in the case of repeat Hb decrease |

CTCAE Version 4.03

**Supplementary Table 2**

Patient characteristics at baseline

|  | **Olaparib**  **(n = 256)** | **Control (n = 131)** |
| --- | --- | --- |
| Median age at randomization (range), years | 69 (47–91) | 69 (49–87) |
| Age ≥65 years at randomization, n (%) | 174 (68) | 97 (74) |
| Metastatic disease at initial diagnosis, n (%) | 66 (26) | 25 (19) |
| Missing data | 11 (4) | 7 (5) |
| Gleason score ≥ 8, n/N (%)^a^ | 183/251 (73) | 95/127 (75) |
| Median PSA at baseline (IQR), μg/L | 68.2 (24.1–294.4) | 106.5 (37.2–326.6) |
| Median Hb, g/dL | 12.3 | 11.9 |
| Measurable disease at baseline^b^, n (%) | 149 (58) | 72 (55) |
| Metastases at baseline^b^, n (%)  Bone only  Visceral (lung/ liver)  Other | 86 (34)  68 (27)  88 (34) | 38 (29)  44 (34)  41 (31) |
| ECOG performance status, n (%)  0  1  2 | 131 (51)  112 (44)  13 (5) | 55 (42)  71 (54)  4 (3) |
| Prior next-generation hormonal agent, n (%)^c^  Enzalutamide only  Abiraterone only  Abiraterone + enzalutamide | 105 (41)  100 (39)  51 (20) | 54 (41)  54 (41)  23 (18) |
| Previous taxane use, n (%)  Docetaxel only  Cabazitaxel only  Docetaxel + cabazitaxel  Paclitaxel only | 170 (66)  115 (45)  3 (1)  51 (20)  1 (<1) | 84 (64)  58 (44)  0  26 (20)  0 |

Hb, haemoglobin; ECOG, Eastern Cooperative Oncology Group; IQR, interquartile range; NHA, next-generation hormonal agent; n/N, no./total no.; PSA, prostate-specific antigen.

^a^ In general, scores on the Gleason scale range from 6 to 10, with higher scores indicating a worse prognosis; ^b^ Data were derived from electronic case-report forms as assessed by the investigator; ^c^ In total, 13 patients received a next-generation hormonal agent (NHA) for disease before a diagnosis of mCRPC; all others received a NHA after the development of mCRPC.

**Supplementary Table 3**

Summary of exposure data, baseline bone metastases and anaemia AEs across olaparib pivotal studies

|  | **POLO (pancreas)**  **n = 91^1^** | **SOLO1 (ovarian)**  **n = 260^2^** | **SOLO2**  **(ovarian) n = 195^3^** | **SOLO3**  **(ovarian)**  **n = 178^4^** | **OlympiAD (breast)**  **n = 205^5^** | **PROfound**  **(prostate) n = 256^6^** |
| --- | --- | --- | --- | --- | --- | --- |
| Study population | BRCA alteration, in response to first-line PBC | BRCA alteration, in response to first-line PBC | BRCA alteration, ≥2 lines PBC, in response to most recent line | BRCA alteration, ≥2 lines PBC,  relapsed | BRCA alteration,  ≤2 lines chemotherapy | HRR alteration, progressed on NHA treatment |
| Median duration of exposure (months) | 6.0 | 24.6 | 19.4 | 11.3 | 8.2 | 7.6 |
| Bone metastases at baseline (%) | 2.2 | 0.8 | 1.0 | N/A | 7.8 | 34.0 |
| Anaemia (all grades) (%) | 27 | 39 | 43.6 | 51.1 | 40.0 | 50 |
| Anaemia (Grade ≥3) (%) | 11 | 22 | 19.5 | 21.3 | 16.1 | 23 |

N/A, Incidence of bone metastases at baseline was not available due to low frequency
HRR, homologous recombination repair; NHA, next-generation hormonal agent; PBC, platinum-based chemotherapy

*Accompanying text to Supplementary Table 3*

With respect to the amount of bone metastases at baseline in PROfound, when other pivotal olaparib studies in different solid tumours to prostate cancer are considered, it can be seen that in PROfound there was a higher proportion of patients with bone metastases at baseline (34%) than in the other studies. Only OlympiAD had a meaningful proportion of patients (7.8%) with bone metastases at baseline. The incidence of anaemia at all grades in the PROfound study is similar to SOLO2 and SOLO3, which are both also studies in later line treatment, and slightly higher than SOLO1, OlympiAD and POLO. The median duration of exposure was also similar between OlympiAD and PROfound and despite the higher frequency of bone metastases at baseline, the incidence of anaemia remained similar. The incidence of Grade ≥3 anaemia is also consistent between all of these pivotal studies at approximately 20% with exception of POLO (11.0%). The similarity in anaemia events across these different olaparib studies suggests that the extent of bone metastases at baseline does not impact the development of anaemia in patients treated with olaparib.

**References**

1. Golan T, Hammel P, Reni M *et al.* Maintenance olaparib for germline BRCA-mutated metastatic pancreatic cancer. *N Engl J Med* 2019;381:317-27.

2. Moore K, Colombo N, Scambia G *et al.* Maintenance olaparib in patients with newly diagnosed advanced ovarian cancer. *N Engl J Med* 2018;379:2495-505.

3. Pujade-Lauraine E, Ledermann JA, Selle F *et al.* Olaparib tablets as maintenance therapy in patients with platinum-sensitive, relapsed ovarian cancer and a BRCA1/2 mutation (SOLO2/ENGOT-Ov21): a double-blind, randomised, placebo-controlled, phase 3 trial. *Lancet Oncol* 2017;18:1274-84.

4. Penson RT, Valencia RV, Cibula D *et al.* Olaparib versus nonplatinum chemotherapy in patients with platinum-sensitive relapsed ovarian cancer and a germline brca1/2 mutation (SOLO3): a randomized phase III trial. *J Clin Oncol* 2020;38:1164-74.

5. Robson M, Im SA, Senkus E *et al.* Olaparib for metastatic breast cancer in patients with a germline BRCA mutation. *N Engl J Med* 2017;377:523-33.

6. Hussain M, Mateo J, Fizazi K *et al.* Survival with olaparib in metastatic castration-resistant prostate cancer. *N Engl J Med* 2020;383:2345-57.
